# Surgical Treatment for Endometrial Cancer, Hysterectomy Performed via Minimally Invasive Routes Compared with Open Surgery: A Systematic Review and Network Meta-Analysis

**DOI:** 10.3390/cancers16101860

**Published:** 2024-05-13

**Authors:** Purushothaman Natarajan, Gayathri Delanerolle, Lucy Dobson, Cong Xu, Yutian Zeng, Xuan Yu, Kathleen Marston, Thuan Phan, Fiona Choi, Vanya Barzilova, Simon G. Powell, James Wyatt, Sian Taylor, Jian Qing Shi, Dharani K. Hapangama

**Affiliations:** 1 Department of Women’s & Children’s Health, Institute of Life Course and Medical Sciences, University of Liverpool, Liverpool L8 7SS, UK; 2 Liverpool Women’s Hospital NHS Foundation Trust, Liverpool L8 7SS, UK; 3 Institute of Applied Health Research, College of Medicine, University of Birmingham, Vincent Drive, Edgbaston B15 2TT, UK; 4 Department of Statistics and Data Science, Southern University of Science and Technology, Shenzhen 518055, China; 5 National Center for Applied Mathematics Shenzhen, Shenzhen 518038, China

**Keywords:** laparoscopy, minimally invasive surgery, minimal access surgery, oncological outcomes, robotic surgery, staging, surgical outcomes

## Abstract

**Simple Summary:**

Keyhole surgery has replaced open surgery as the gold standard of care in the surgical treatment of cancer of the womb. Previous reviews comparing keyhole and open surgery exclusively analysed data from randomised control trials. We present a comprehensive review using randomised and non-randomised trials to compare keyhole surgery and open surgery. This review investigates benefits, complications and long-term outcomes in terms of survival after treatment of cancer of the womb, and it shows that keyhole surgery lessened blood loss and the length of hospital stay compared to open surgery. Among the keyhole methods, robotic surgery decreased some complications while rendering the return of cancer less likely.

**Abstract:**

**Background**: Total hysterectomy with bilateral salpingo-oophorectomy via minimally invasive surgery (MIS) has emerged as the standard of care for early-stage endometrial cancer (EC). Prior systematic reviews and meta-analyses have focused on outcomes reported solely from randomised controlled trials (RCTs), overlooking valuable data from non-randomised studies. This inaugural systematic review and network meta-analysis comprehensively compares clinical and oncological outcomes between MIS and open surgery for early-stage EC, incorporating evidence from randomised and non-randomised studies. **Methods**: This study was prospectively registered on PROSPERO (CRD42020186959). All original research of any experimental design reporting clinical and oncological outcomes of surgical treatment for endometrial cancer was included. Study selection was restricted to English-language peer-reviewed journal articles published 1 January 1995–31 December 2021. A Bayesian network meta-analysis was conducted. **Results**: A total of 99 studies were included in the network meta-analysis, comprising 181,716 women and 14 outcomes. Compared with open surgery, laparoscopic and robotic-assisted surgery demonstrated reduced *blood loss* and *length of hospital stay* but increased *operating time*. Compared with laparoscopic surgery, robotic-assisted surgery was associated with a significant reduction in *ileus* (OR = 0.40, 95% CrI: 0.17–0.87) and *total intra-operative complications* (OR = 0.38, 95% CrI: 0.17–0.75) as well as a higher *disease-free survival* (OR = 2.45, 95% CrI: 1.04–6.34). **Conclusions**: For treating early endometrial cancer, minimal-access surgery via robotic-assisted or laparoscopic techniques appears safer and more efficacious than open surgery. Robotic-assisted surgery is associated with fewer complications and favourable oncological outcomes.

## 1. Introduction

Endometrial cancer (EC) is the sixth most common cancer in women and the 15th most common cancer overall. Its worldwide incidence is 8.7/10,000 (Age-standardized rate), with more than 417,000 new cases diagnosed in 2020 [1]. EC risk increases with age, and the highest rates are reported in women aged 75–79 [2]. A European Cancer Registry study demonstrated an overall survival of 76% in women diagnosed with EC in the years 2000–2007 [3].

EC typically presents early with postmenopausal bleeding but may also present with persistent intermenstrual and heavy vaginal bleeding associated with features of anovulation [4]. Diagnosis is accomplished through a histological evaluation of an endometrial sample, and surgery is the first-line treatment. The extent of surgery depends on histopathological features such as type, grade and International Federation of Gynaecology and Obstetrics (FIGO) stage. For early-stage EC (FIGO stage 1–2), total hysterectomy with bilateral salpingo-oophorectomy (BSO) is the standard treatment [5], and minimally invasive surgery (MIS) has become the preferred mode. Sentinel node dissection and omental biopsy may be considered in high-risk disease. In stage 2 disease, total hysterectomy with BSO is adequate, but a radical hysterectomy may be required to achieve margin-free resection. Exclusive open surgery (OS) is advocated in advanced disease (FIGO stages 3 and 4), where primary debulking surgery can be considered if associated morbidity and quality of life are acceptable. Palliative surgery also has a role in symptomatic women with advanced EC [5].

The rate of EC is estimated to be increasing, and as the awareness of associated symptoms increases, most women are expected to present with early stages of cancer, for which surgical treatment is often curative. The surgical management of EC, therefore, is an important area of clinical care and research. Current guidelines recommend MIS as the preferred route for early-stage EC (FIGO Stage 1 and 2) based on evidence from randomised control trials (RCTs) demonstrating low post-operative morbidity with comparable oncological outcomes [5,6]. These recommendations are associated with the widespread adoption of MIS in clinical practice. A recent Cochrane review has shown that laparoscopic surgery (LRS) is associated with similar overall survival and recurrence rates with reduced post-operative morbidity. Quality of life (QOL) was better in the LRS group for the first three years; however, after four years, QOL was similar in both groups [7]. RCTs have also demonstrated robotic surgery (RS) to be non-inferior to either standard LRS or OS; however, the available evidence is limited on long-term outcomes of the RS approach [5,8,9,10,11].

RCTs typically only report a handful of possible risks and outcomes. Further useful clinical information may be available from non-randomised studies. In this regard, there have not been sufficient attempts to capture all reported risks associated with the different surgical approaches available for hysterectomy indicated in early-stage EC, which includes evidence from non-randomised studies. Such data help patients with EC and their clinicians in shared decision making regarding surgical treatment while also informing healthcare providers to align their services.

With this background, our aim was to systematically collate the published evidence to determine the comparative surgical and oncological outcomes related to three different surgical treatment options for early stages of EC. Therefore, we systematically reviewed the published evidence from randomised and non-randomised studies reporting clinical and oncological outcomes of both MIS (laparoscopic or robotic) and OS in treating early-stage EC. We believe this will facilitate best practice in shared, informed decision making and the process of consent in the surgical treatment of early-stage EC.

## 2. Materials and Methods

### 2.1. Eligibility Criteria, Information Sources, Search Strategy

This study followed the PRISMA statement for systematic reviews and meta-analyses. The study protocol was prospectively registered with PROSPERO (CRD42020186959). A systematic literature search was conducted using PubMed (https://pubmed.ncbi.nlm.nih.gov/), EMBASE (https://www.wolterskluwer.com/en/solutions/ovid/ovid-medline-901), Science Direct (https://www.sciencedirect.com/) and the ISRCTN registry (https://www.wolterskluwer.com/en/solutions/ovid/embase-903), all last accessed on 29 October 2020. The use of MIS for treating EC is relatively recent [12], with the first RCT published in 2002 [13]. Thus, considering the need for at least three years of follow-up to complete long term oncological outcomes, the search was conducted to obtain all clinically relevant data from peer-reviewed, published studies conducted between 1 January 1995 and 31 December 2021. Following duplicate deletion, titles and abstracts were screened by one author (PN) to assess eligibility for full review. The full-text review papers were then evaluated by multiple authors (PN, LD, TP, KM, VB, FC), and the discrepancies were resolved through consensus discussion. Search results were supplemented with the forward and backward chaining of the references for the included studies.

### 2.2. Study Selection

All peer-reviewed and published studies, including randomised and non-randomised studies that reported outcomes for patients with early-stage EC undergoing a hysterectomy via RS, LRS or OS, were included. All studies reporting hysterectomy for endometrial hyperplasia, benign gynaecological conditions or non-endometrial cancer and studies reported in any other language but English were excluded.

### 2.3. Data Extraction

Data screening and extraction were completed in line with the inclusion and exclusion criteria by three independent reviewers. Effect sizes, odds ratios (OR), sample size, complication type and geographical location were extracted and developed within a specific data extraction template. All complication details identified within each study were then coded and defined prior to completing the statistical analyses.

### 2.4. Risk of Bias of Included Studies

The Newcastle Ottawa Quality Assessment Scale (NOS) was used to assess the risk of bias. The studies were evaluated using the following criteria: selection, comparability and exposure. A maximum of four stars was awarded for selection, two for comparability and three for outcomes, with a maximum of nine stars. NOS was used to assess the quality of both randomised and non-randomised studies. The studies were categorised into low risk if they scored 7–9 stars, moderate risk if they scored 5–6 stars and high risk if they scored 0–4 stars. Fifteen studies were in the high-risk group, and the majority (80) were in the low-risk group.

### 2.5. Synthesis of Results

The primary outcomes extracted from the studies include *duration of operation, length of stay in hospital,* intra-operative complications (e.g., *blood loss*), incidence of additional treatments (e.g., blood transfusion), post-operative complications (e.g., *fever, infection, ileus*), complications of uncertain timing (e.g., *VTE*), *total complications, total intraoperative* and *post-operative complications* and oncological outcomes of *disease-free survival* and *recurrence*.

Statistical analyses were undertaken on R version 4.0.2, with the packages “metafor”, “rjags”, and “gemtc”, and RStudio version 1.3.1073.

The ten binary outcomes (blood transfusion, fever, infection, ileus, VTE, total intra-operative complications, total complications, total post-operative complication, recurrence and disease-free survival) were assessed and reported with OR and the corresponding 95% credibility interval (CrI) calculated from absolute numbers or percentages. The four continuous outcomes (blood loss, duration of operating time, length of hospital stay and total number of lymph nodes dissected) were assessed and reported with mean difference (MD) and their computed variances. For studies where only median values and ranges (or interquartile ranges) of continuous outcomes were reported, the results were transformed into means and variances [14].

The network meta-analysis (NMA), a random-effect model, was expanded with a Bayesian method that allowed the inclusion of direct and indirect comparisons of the surgical techniques used, allowing for a better understanding of the data [15,16,17]. The simultaneous inference of the evidence, considering the three surgical interventions, was facilitated by a data structure that could be regarded as a k-comparison to synthesise the available evidence. In line with this, the research question: “*What is the prevalence of complications associated with three surgical techniques used among EC patients*?” was developed and answered via the following distinct aims. The prevalence of peri-operative and oncological complications associated with RS versus LRS versus OS, as well as their rating, assessment of performance and clinical effectiveness defined by the rate of complications associated with each surgical method, were investigated. The Markov Chain Monte Carlo (MCMC) simulation was applied to estimate the posterior distributions of the model parameters and generate the results. The convergence of the MCMC process was assessed by evaluating the trace plots, and the consistency assumption was checked by performing a node-split analysis, which evaluates every comparison of interest using a separate model [18].

For the interpretation of the Bayesian NMA results, Forest plots, Rankograms and surface under the cumulative ranking (SUCRA) plots were used [19,20]. Based on an interim empirical evaluation of the last decade, a trend of moving away from OS to LRS and RS was observed within the pooled studies. As a result, to assess the possible time trend of the outcomes, a meta-regression of each identified outcome based on the time period of the study was performed. The study year of all publications included was utilised as the midpoint of the study duration using the following formula:

Study year = (study start year + study end year)/2

The estimated regression coefficients of the study years were examined to report time trends based on current empirical evidence reported from practitioners.

## 3. Results

### 3.1. Study Characteristics

**Figure 1:**
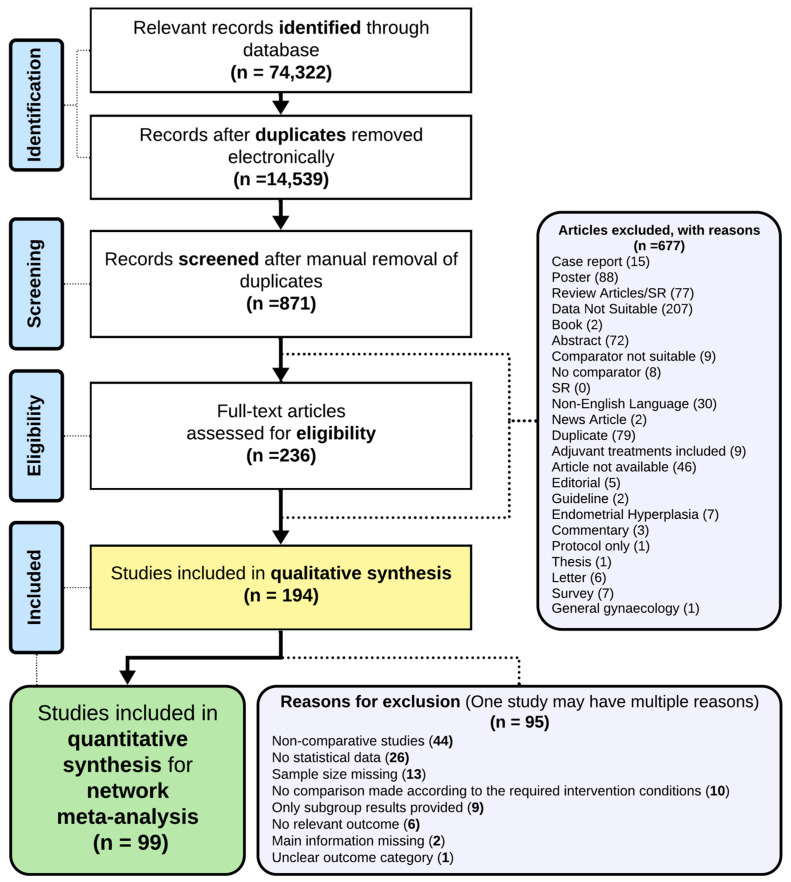
The PRISMA (Preferred Reporting Items for Systematic Reviews and Meta-analyses) diagram.

The initial search yielded 74,322 references, from which 59,783 duplicates were removed, and the remaining 14,539 records were screened to select 874 relevant publications to assess the abstracts for eligibility. A total of 194 studies [9,13,21,22,23,24,25,26,27,28,29,30,31,32,33,34,35,36,37,38,39,40,41,42,43,44,45,46,47,48,49,50,51,52,53,54,55,56,57,58,59,60,61,62,63,64,65,66,67,68,69,70,71,72,73,74,75,76,77,78,79,80,81,82,83,84,85,86,87,88,89,90,91,92,93,94,95,96,97,98,99,100,101,102,103,104,105,106,107,108,109,110,111,112,113,114,115,116,117,118,119,120,121,122,123,124,125,126,127,128,129,130,131,132,133,134,135,136,137,138,139,140,141,142,143,144,145,146,147,148,149,150,151,152,153,154,155,156,157,158,159,160,161,162,163,164,165,166,167,168,169,170,171,172,173,174,175,176,177,178,179,180,181,182,183,184,185,186,187,188,189,190,191,192,193,194,195,196,197,198,199,200,201,202,203,204,205,206,207,208,209,210,211,212,213,214], with 245,408 women, were included in the review (Table 1), and out of them, a total of 99 [9,23,24,26,30,32,36,39,42,43,47,49,51,52,53,54,56,57,59,63,67,68,70,71,76,77,78,79,81,82,84,85,87,88,89,90,94,96,100,102,105,110,111,112,113,114,119,122,123,124,128,132,134,135,136,138,139,143,145,153,154,159,164,165,167,168,169,170,171,172,173,177,178,180,181,182,183,184,186,187,188,190,192,194,195,196,198,200,201,202,208,209,210,212,213,214,215,216,217,218,219], comprising five RCTs and 94 cohort studies, were included in the NMA. The types of studies are detailed in the study characteristics and included 181,716 women. The PRISMA (Preferred Reporting Items for Systematic Reviews and Meta-analyses) diagram (Figure 1) illustrates the process of elimination. Detailed characteristics and a quality analysis of a subset of the studies included in the current systematic review and meta-analysis are listed in Table 1. The NMA utilised 14 outcomes, while those for other outcomes reported in fewer than ten studies were omitted (Table 2).

### 3.2. Intra-Operative Outcomes (Figure 2 and Figure 3, Table 2, Table 3 and Table 4)

#### 3.2.1. Blood Loss

Compared with OS, LRS and RS demonstrated statistically significant differences of −226.90 millilitre (mL) (95% CrI: −298.40–−155.90) and −257.20 mL (95% CrI: −351.20–−163.80) of *Blood loss*, respectively. This suggests that patients undergoing LRS or RS had significantly less blood loss than those undergoing OS. However, the difference between RS and LRS was not statistically significant, with an MD of −30.33 (95% CrI: −122.2–61.62).

**Table 3:** SUCRA (surface under the cumulative ranking) scores of the three surgical techniques (LRS: laparoscopic surgery, OS: open surgery, RS: robotic surgery) for the 14 outcomes from the Bayesian network meta-analysis. The significant differences are shown in bold.

Outcome	OS	LRS	RS
**Blood Loss**	0.0000	0.6275	**0.8725**
**Duration of Operation**	**0.9996**	0.4496	0.0508
**Length of Stay in Hospital**	0.0000	0.6576	**0.8424**
**Total Lymph Nodes**	**0.5924**	0.3200	0.5877
**Blood Transfusion**	0.0002	0.6661	**0.8338**
**Fever**	0.0783	0.6683	**0.7533**
**Infection**	0.1757	**0.9319**	0.3925
**Ileus**	0.0005	0.5055	**0.9941**
**VTE**	0.1567	**0.8688**	0.4745
**Disease-free Survival**	0.0616	0.4509	**0.9876**
**Recurrence**	0.0393	**0.7606**	0.7001
**Total Complications**	0.0000	0.6194	**0.8806**
**Total Intra-operative Complications**	0.3017	0.2014	**0.9969**
**Total Post-operative Complications**	0.0014	0.7118	**0.7868**

**Table 4:** Results of meta-regressions on study year for the 14 outcomes. The significant differences are demonstrated in bold.

Outcome		k	β	SE of β	*p*-Value
**Blood Loss**	LRS vs. OS	41	1.2784	9.7550	0.8957
	RS vs. OS	16	−6.7529	17.6153	0.7015
	**RS vs. LRS**	**18**	**15.8981**	**4.3915**	**0.0003**
**Duration of Operation**	**LRS vs. OS**	**39**	**−2.3569**	**1.1987**	**0.0493**
	RS vs. OS	15	−4.9162	3.2948	0.1357
	RS vs. LRS	14	0.6972	3.1650	0.8257
**Length of Stay in Hospital**	LRS vs. OS	44	0.0241	0.0702	0.7317
	RS vs. OS	13	0.0773	0.2957	0.7939
	RS vs. LRS	13	0.1858	0.1794	0.3003
**Total Lymph Nodes**	LRS vs. OS	12	−0.3310	0.2225	0.1368
**Blood Transfusion**	**LRS vs. OS**	**19**	**−0.0915**	**0.0380**	**0.0160**
**Fever**	LRS vs. OS	10	0.0057	0.0699	0.9344
**Infection**	LRS vs. OS	16	−0.0395	0.0525	0.4523
**Disease-free Survival**	LRS vs. OS	11	0.0098	0.0262	0.7087
**Recurrence**	LRS vs. OS	20	0.0130	0.0334	0.6983
**Total Complications**	LRS vs. OS	24	0.0414	0.0213	0.0526
	**RS vs. LRS**	**11**	**0.1415**	**0.0674**	**0.0357**
**Total Intra-operative Complications**	LRS vs. OS	14	−0.0448	0.0378	0.2369
**Total Post-operative Complications**	LRS vs. OS	20	−0.0058	0.0438	0.8950

**Figure 2:**
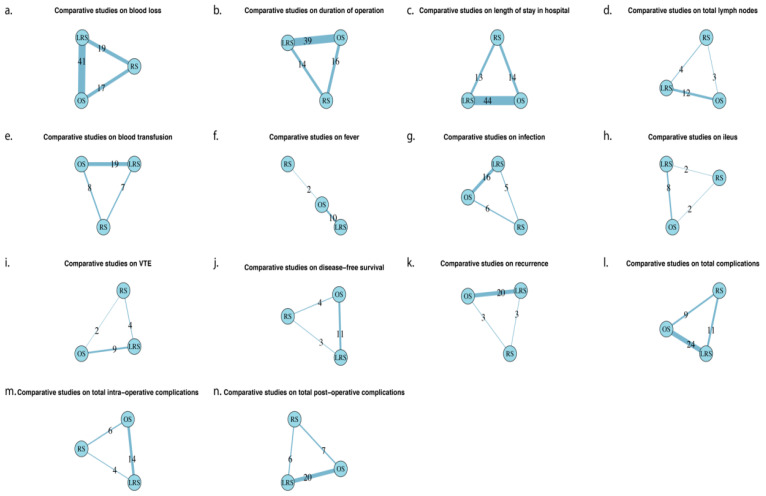
Results of the node-split analysis checking consistency and assumptions. The effect sizes and 95% credible intervals from direct comparison, indirect comparison and the network combining the two are shown in Figure 2. The *p*-values in this context were used to test the consistency between direct and indirect comparisons. Figure 2 demonstrates that the consistency assumption is generally satisfied for 11 outcomes. The remaining three outcomes, fever, disease-free survival and total-intraoperative complications, were not shown due to insufficient data. (LRS: laparoscopic surgery, OS: open surgery, RS: robotic surgery).

**Figure 3:**
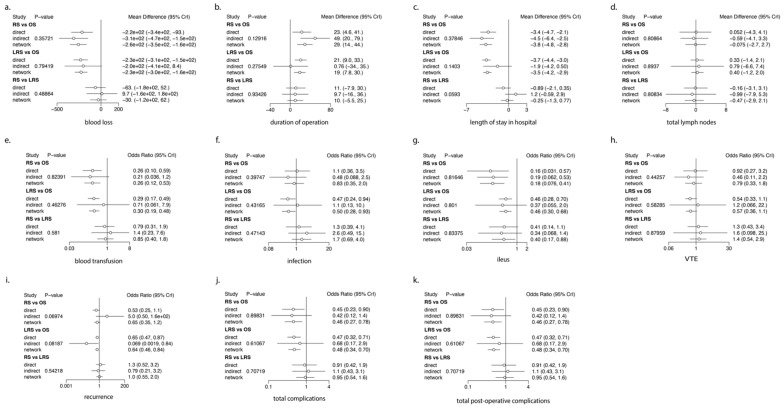
Shows the forest plots of the 14 outcomes, providing the pooled estimates of the effect size of each surgery technique compared to open surgery (OS). (LRS: laparoscopic surgery, OS: open surgery, RS: robotic surgery).

#### 3.2.2. Duration of Operating Time

There were statistically significant increases in the *duration of operating time* of 18.95 min (95% CrI: 7.68–30.20) with LRS and 29.00 min (95% CrI: 13.66–44.23) with RS compared with OS, respectively. This suggests that patients undergoing LRS or RS had a significantly longer duration of operation than those undergoing OS. The difference between RS and LRS is not statistically significant (MD = 10.05 min, 95% CrI: −5.60–25.48).

#### 3.2.3. Total Lymph Nodes Resected

There was no statistically significant difference for LRS and RS, with mean differences of 0.4 (95% CrI: −1.18–2.01) and −0.06 (95% CrI: −2.91–2.69) compared with OS, respectively. Furthermore, the difference between RS and LRS is not statistically significant, with an MD of −0.46 (95% CrI: −2.91–2.09).

### 3.3. Post-Operative Outcomes (Figure 2 and Figure 3, Table 2, Table 3 and Table 4)

For the comparison between LRS and OS, there were statistically significant differences in the following post-operative binary outcomes: *blood transfusion* (OR = 0.30, 95% CrI: 0.19–0.48), *fever* (OR = 0.57, 95% CrI: 0.30–0.98), *infection* (OR = 0.50, 95% CrI: 0.28–0.93), *ileus* (OR = 0.46, 95% CrI: 0.29–0.68), *total complications* (OR = 0.38, 95% CrI: 0.29, 0.51), *total post-operative complications* (OR = 0.48, 95% CrI: 0.34–0.70). These results suggest that patients undergoing LRS had a significantly lower incidence of *blood transfusion*, *fever*, *infection*, *ileus*, *recurrence*, *total complications* and *total post-operative complications* than those undergoing OS. On the other hand, the incidence of *VTE*, *disease-free survival* and *total intra-operative complications* were not significantly different between LRS and OS.

Comparing RS with OS, there were statistically significant differences in the following binary post-operative outcomes: *blood transfusion* (OR = 0.26, 95% CrI: 0.12–0.53), *ileus* (OR = 0.18, 95% CrI: 0.08–0.41), *total complications* (OR = 0.34, 95% CrI: 0.22–0.51), *total intra-operative complications* (OR = 0.39, 95% CrI: 0.18–0.78), *total post-operative complications* (OR = 0.46, 95% CrI: 0.27–0.78). These results suggest that patients undergoing RS had a significantly lower incidence of *blood transfusion*, *ileus*, *total complications*, *total intra-operative complications* and *total post-operative complications* and better *disease-free survival* than those undergoing OS. On the other hand, the incidence of *fever*, *infection* and *VTE* was not significantly different between RS and OS.

When RS was compared with LRS, there were statistically significant differences in two binary post-operative outcomes: *ileus* (OR = 0.40, 95% CrI: 0.17–0.87) and *total intra-operative complications* (OR = 0.38, 95% CrI: 0.17–0.75). These results suggest that patients undergoing RS had a significantly lower incidence of *ileus* and *total intra-operative complications* than those undergoing LRS. The incidence of other binary outcomes was not significantly different between RS and LRS.

#### Length of Hospital Stay

Compared with OS, there was a statistically significant reduction in the length of hospital stay in women who underwent LRS and RS with mean differences of −3.54 days (95% CrI: −4.22–−2.87) and −3.79 days (95% CrI: −4.79–−2.79), respectively. This suggests that patients undergoing MIS had a significantly shorter length of stay in hospital than those undergoing OS. The difference between RS and LRS is not statistically significant (MD = −0.25 days, 95% CrI: −1.26–0.77).

### 3.4. Oncological Outcomes (Figure 2 and Figure 3, Table 2, Table 3 and Table 4)

There was a significant reduction in the binary outcomes of cancer *recurrence* (OR = 0.64, 0.47–0.84) with LRS compared to OS. The incidence of *disease-free survival* was not significantly different between LRS and OS.

When RS was compared with OS, there was a significantly higher *disease-free survival* (OR = 3.29, 95% CrI: 1.46–8.36) associated with this method, but *recurrence* was not significantly different between RS and OS.

Compared with LRS, RS was associated with significantly higher *disease-free survival* (OR = 2.45, 95% CrI: 1.04–6.34), but the other oncology outcomes appear to be similar between the two approaches.

### 3.5. Ranking Analysis (Figure 2 and Table 2 and Table 3)

Ranking analysis indicates that OS is the best technique when the *duration of operation* or *total lymph nodes* are considered, LRS is the best technique when incidences of *infection*, *VTE* and *recurrence* are considered, and RS is the best technique when *blood loss*, *length of stay in hospital*, *disease-free survival* and incidences of *blood transfusion*, *fever*, *ileus*, *total complications*, *total intra-operative complications* and *total post-operative complications* are considered.

### 3.6. Meta-Regression Analysis (Figure 4 and Table 4)

A meta-regression analysis was conducted for each outcome in line with the study timelines to study the possible time trend on the outcomes.

**Figure 4:**
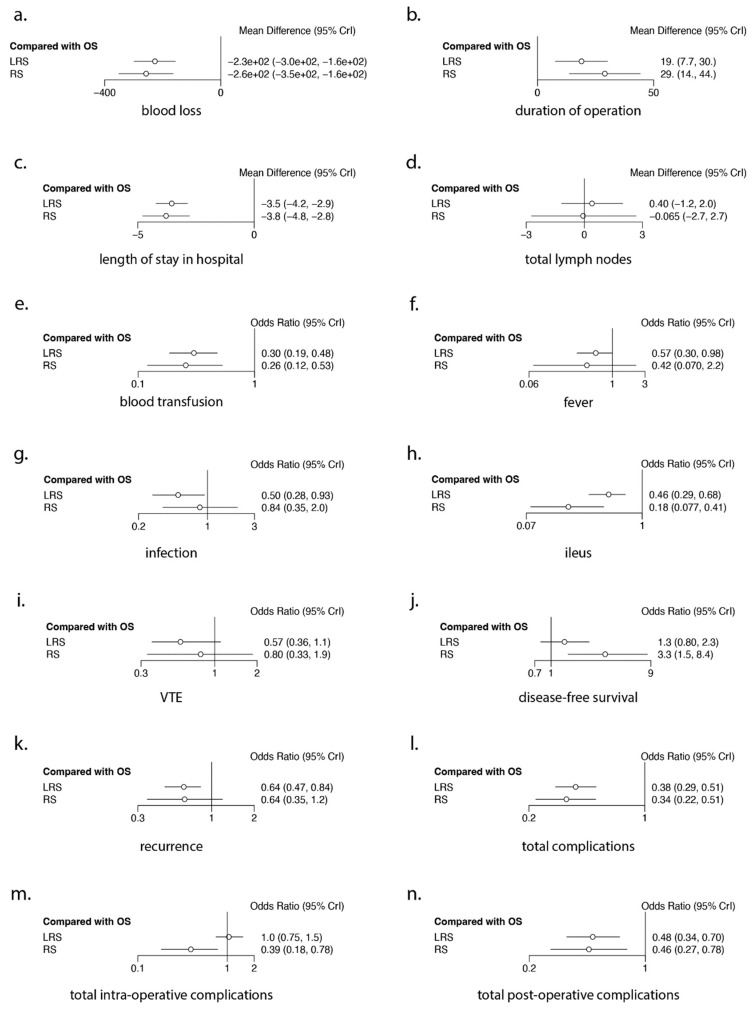
Forest plots of the Bayesian network meta-analyses for each of the 14 outcomes.

The study year did not significantly affect comparisons among the three surgical techniques on most outcomes. However, time trend was significant in four cases: comparison between LRS vs. OS on *duration of operation* (estimated regression coefficient −2.3596 (*p* = 0.0493)), comparison between RS vs. LRS on *blood loss* (estimated regression coefficient 15.8981 (*p* = 0.0003)), comparison between LRS vs. OS on *blood transfusion* (estimated regression coefficient −0.0915 (*p* = 0.0160)) and comparison between RS vs. LRS on *total complications* (estimated regression coefficient 0.1415 (*p* = 0.0357)) (Figure 4).

These data suggest that some differences between techniques appear to reduce in magnitude with time. For example, the initial longer *duration of operation* between LRS and OS became smaller over time. Similarly, the difference in *blood loss* between RS and LRS also reduced over time.

Conversely, the difference in a lower incidence of *blood transfusion* between LRS and OS increased over time. Although the earlier studies reported a lower incidence of *total complications* in patients undergoing RS vs. LRS, the more recent studies reported contrastingly lower *total complication* rates with LRS than RS.

## 4. Discussion

### 4.1. Principal Findings

#### 4.1.1. Robotic-Assisted Surgery

The superiority of RS in *disease-free survival*, when compared with either OS or LRS, is of key interest. RS also demonstrated improved peri-operative outcomes, including a reduction in *blood loss*, *length of stay in hospital*, *total intra-operative complications* and incidences of *blood transfusion*, *fever*, *ileus*, *total post-operative complications* and *total complications* according to the ranking analysis. However, the recommendation of RS as the best option for hysterectomy for early EC should be made with caution since the meta-regression analysis demonstrated a possible increase in *total complications* associated with the RS route compared to LRSs. There has been an increasing number of surgeons learning and performing hysterectomies via the RS route in recent years as opposed to the highly experienced specialists as in early studies, and thus increasing numbers of RS cases may explain these findings. Nevertheless, future studies should focus on further examining this trend.

#### 4.1.2. Laparoscopic Surgery

The LRS approach appears to be the best technique to consider when the reduction of incidences of *infection*, *VTE*, and *recurrence* is desired according to the ranking analysis. However, OS faired best when reducing the *duration of operation* or increasing the *total number of lymph nodes to be harvested.* This information is vital for service planning and future directions in the management of EC. The main disadvantage of LRS is that the duration of surgery appears to reduce with time, according to our meta-regression analysis, possibly due to increasing skills in LRS.

### 4.2. Comparison with Existing Literature

This study presents a comprehensive systematic review including 194 manuscripts and an NMA comprising 99 papers, demonstrating the significant superiority of MIS for early-stage EC, compared with OS in multiple aspects. Although the *duration of surgery* was slightly longer, MIS was associated with significantly lower rates of complications during and after surgery, in conjunction with a possible superiority in oncological outcomes, compared with those who underwent OS. MIS approaches were also associated with a reduced *duration of hospital stay*, which relates to reduced health service costs. Our findings are consistent with the Cochrane review [7] on LRS for EC and large RCTs such as LACE [21] and LAP2 [22]. OS was associated with a higher incidence of post-operative complications such as *Fever, infection* and *ileus* compared with MIS. Previous Cochrane reviews and other RCTs [23] have shown comparable rates of *recurrence* and *disease-free survival* with the OS or MIS approach, yet our review has shown that MIS (LRS or RS) is associated with lower *recurrence* and better *disease-free survival*. The reasons for these observations are unclear but may be due to increasing expertise in MIS over recent years and possible selection bias, where high-risk ECs were preferentially treated by OS. However, with the recent advent of robotic surgery, high-risk patients, for example, with morbid obesity have been particularly assigned to undergo surgery via MIS route, and thus this data may be a true reflection of superior outcomes.

### 4.3. Strengths and Limitations

The main strength of this study is the inclusion of all relevant published data from both RCTs and cohort studies. By including data from cohort studies that were excluded by previous systematic reviews, our study represents, to our knowledge, the most comprehensive summary to date of peri- and post-operative and oncological outcomes associated with surgical treatment for early EC. This data thus could provide a solid foundation for developing core outcomes for hysterectomy for EC. The inclusion of observational data limits causal inferences and inevitably is subjected to carryover confounding bias. Non-standardised outcome reporting limited the number of studies that could be included in the analysis. We did not formally investigate selection bias.

It is also important to note that trends in endometrial cancer staging surgery have changed over time (with improved pre-operative imaging and molecular subtyping influencing the extent of surgery [6]). In particular, the advent of sentinel lymph node biopsy has resulted in far fewer systematic pelvic and para-aortic lymph node dissections, thus reducing associated risks and morbidity. However, this practice varies considerably between institutions. Facilities for sentinel lymph node biopsy are not universally available, and not all institutions have the expertise to perform laparoscopic lymph node dissections. These variations in practice and techniques (e.g., new minimal access approaches including natural orifice transvaginal endoscopic surgery (NOTES), laparo-endoscopic single site (LESS) surgery and robotic single-site surgery) will introduce heterogeneity to the studies. Therefore, discussion regarding the risks of surgery should be tailored to the patient, surgeons and the cancer unit, considering the anticipated complexity of the planned operation and locally available expertise and resources when assessing the relevance of the outcomes reported here. The majority of studies included compared outcomes for different surgical approaches within the same institution(s), and we have included a large number of studies from a large geographical area. Thus, it is reasonable to assume heterogeneity within each surgical approach for the findings to be generalisable.

## 5. Conclusions

MIS, via either the robotic or the laparoscopic route, appears to be a safer and more efficacious approach when compared with OS for the treatment of early EC. The MIS approach is associated with fewer complications with favourable oncological outcomes. Time trends on outcomes, identified in our meta-regression analysis, provide vital information for policymakers and researchers to use in future-proofing health services and clinical trials.

## Figures and Tables

**Table 1:** Characteristics of the 194 studies included in the qualitative synthesis.

Author	Year of Study	Sample Size	Type of Study	Comparator
Abel et al. [21]	2020	1805	Retrospective Cohort Study	Laparoscopic
Abitbol et al. [22]	2016	340	Retrospective Cohort Study	Robotic
Agarwal et al. [23]	2018	133	Retrospective Cohort Study	Robotic/Open
Aiko et al. [24]	2020	223	Retrospective Cohort Study	Laparoscopic/Robotic
Ansar et al. [25]	2018	60	Prospective Non-Randomized Control Study	Laparoscopic/Open
Api et al. [26]	2013	79	Retrospective Cohort Study	Laparoscopic/Open
Armfield et al. [27]	2018	404	Randomized Controlled Trial	Laparoscopic/Open
Avondstond et al. [28]	2017	40	Retrospective Cohort Study	Robotic
Backes et al. [29]	2015	543	Retrospective Cohort Study	Robotic
Backes et al. [30]	2016	182	Retrospective Cohort Study	Robotic/Open
Baek et al. [31]	2014	278	Retrospective Cohort Study	Laparoscopic
Bajaj et al. [32]	1999	70	Retrospective Cohort Study	Laparoscopic/Open
Baker et al. [33]	2013	760	Randomized Controlled Trial	Laparoscopic/Open
Baker et al. [34]	2015	760	Randomized Controlled Trial	Laparoscopic/Open
Bakkum Gamez et al. [35]	2013	1369	Retrospective study	Laparoscopic/Robotic/Open/Vaginal
Ball et al. [36]	2011	289	Retrospective Cohort Study	Laparoscopic/Open
Barber et al. [37]	2016	9948	Retrospective Cohort Study	Minimally Invasive/Open
Barnes et al. [38]	2017	210	Retrospective Cohort study	Laparoscopic
Barnett et al. [39]	2011	376	Retrospective Cohort Study	Laparoscopic/Open
Barnett et al. [40]	2010	0	Decision Model Analysis	Laparoscopic/Robotic/Open
Barraez et al. [41]	2014	446	Retrospective Study	Robotic
Barwijuk et al. [42]	2005	25	Retrospective Study	Laparoscopic/Open
Beck et al. [45]	2018	3712	Retrospective Cohort Study	Laparoscopic/Open
Bell et al. [43]	2008	110	Retrospective Study	Laparoscopic/Robotic/Open
Bennich et al. [44]	2016	227	Retrospective Study	Laparoscopic
Bergstrom et al. [46]	2018	1621	Retrospective Cohort Study	Laparoscopic/Robotic/Vaginal
Bernardini et al. [47]	2012	86	Retrospective Cohort Study	Robotic/Open
Berretta et al. [48]	2015	81	Retrospective Cohort Study	Laparoscopic/Open/Vaginal
Bige et al. [49]	2015	140	Prospective Non-Randomized Control Study	Laparoscopic/Open
Bishop et al. [50]	2018	1477	Randomized Controlled Trial	Laparoscopic/Open
Bogani et al. [51]	2014	125	Retrospective Study	Laparoscopic/Open
Bogani et al. [52]	2016	638	Retrospective Cohort Study	Robotic/Open
Boggess et al. [53]	2008	322	Retrospective Cohort Study	Laparoscopic/Robotic/Open
Boosz [215]	2014	267	Retrospective Study	Laparoscopic/Open
Bourgin et al. [54]	2017	344	Retrospective Study	Laparoscopic/Robotic/Open/Vaginal
Bouwman et al. [55]	2015	514	Retrospective Study	Laparoscopic/Open
Casarin et al. [196]	2020	35,224	Retrospective Study	Laparoscopic/Robotic/Open
Casarin et al. [197]	2018	12,283	Retrospective Cohort Study	Laparoscopic/Robotic/Open
Chan [198]	2015	1087	Retrospective Study	Laparoscopic/Robotic/Open
Chiou et al. [199]	2015	377	Retrospective Study	Robotic
Cho et al. [201]	2007	288	Retrospective Study	Laparoscopic (LAVH)/Laparoscopic (TLH)/Open
Chu et al. [202]	2016	151	Retrospective Cohort Study	Laparoscopic/Open
Chung et al. [200]	2019	15	Retrospective Study	Robotic
Coronado et al. [203]	2012	347	Retrospective Study	Laparoscopic/Robotic/Open
Corrado et al. [205]	2016	50	Retrospective Study	Laparoscopic
Corrado et al. [136]	2015	526	Retrospective Study	Laparoscopic/Open
Corrado et al. [207]	2018	655	Retrospective Study	Laparoscopic/Robotic
Corrado et al. [204]	2018	45	Prospective Cohort Study	Robotic
Corrado et al. [216]	2016	125	Prospective Cohort Study	Robotic
Corrado et al. [206]	2016	70	Retrospective Study	Robotic
Cybulska et al. [208]	2018	760	Retrospective Study	Laparoscopic/Robotic
Dai et al. [209]	2020	519	Retrospective Study	Laparoscopic/Open
DeNardis et al. [210]	2008	162	Retrospective Study	Robotic/Open
Deura et al. [211]	2019	120	Retrospective Study	Laparoscopic/Open
Dietrich et al. [212]	2019	350	Retrospective Study	Minimally Invasive/Open
Dowdy et al. [213]	2012	1369 (of 1415 patients identified)	Retrospective Study	Minimally Invasive/Open
Eltabbakh [62]	2000	75	Retrospective Study	Laparoscopic
Eltabbakh [63]	2002	186	Retrospective Study	Laparoscopic/Open
Fader et al. [68]	2016	32,560	Retrospective Study	Laparoscopic/Open
Fader et al. [67]	2012	383	Retrospective Cohort Study	Laparoscopic/Robotic/Open
Fagotti et al. [69]	2012	100	Retrospective Study	Laparoscopic
Fagotti et al. [70]	2013	57	Retrospective Study	Laparoscopic
Fagotti et al. [71]	2012	150	Retrospective Study	Laparoscopic/Robotic
Fanning et al. [72]	2010	235	Retrospective Study	Laparoscopic
Farthing et al. [73]	2012	191	Retrospective Study	Laparoscopic
Fleming et al. [74]	2012	66	Retrospective Cohort Study	Laparoscopic/Robotic/Open
Fleming et al. [75]	2011	181	Retrospective Cohort Study	Laparoscopic/Robotic/Open
Fram et al. [13]	2002	61	Randomized Controlled Trial	Laparoscopic (LAVH)/Open
Freeman et al. [94]	2016	1433	Retrospective Study	Laparoscopic/Robotic
Frey et al. [95]	2015	122	Retrospective Study	Laparoscopic/Robotic
Frey et al. [58]	2011	129	Retrospective Cohort Study	Laparoscopic/Robotic/Open
Frigerio et al. [96]	2006	110	Retrospective Study	Laparoscopic (LAVH)/Open
Gambacorti-Passerini et al. [97]	2019	83	Prospective Observational Study	Laparoscopic
Gehrig et al. [76]	2008	79	Retrospective Cohort Study	Laparoscopic/Robotic
Gemignani et al. [77]	1999	320	Retrospective Study	Laparoscopic (LAVH)/Open
Ghazali et al. [78]	2019	40	Retrospective Cohort Study	Laparoscopic/Open
Ghezzi et al. [79]	2006	101	Prospective Cohort Study	Laparoscopic
Ghezzi et al. [80]	2006	72	Randomized Controlled Trial	Laparoscopic (LAVH/TLH)
Ghezzi et al. [81]	2009	103	Retrospective Study	Laparoscopic (Microlaparoscopy/Conventional Laparoscopy)
Ghezzi et al. [82]	2010	117	Prospective Cohort Study	Laparoscopic/Open
Giannini et al. [98]	2020	100	Retrospective Study	Laparoscopic/Open
Giannini et al. [83]	2021	147	Retrospective Study	Robotic (DaVinci robot Si/XI)
Gildea et al. [99]	2016	46,859	Retrospective Study	Laparoscopic/Open
Gil-Moreno et al. [84]	2006	370	Retrospective Cohort Study	Laparoscopic/Open
Giray et al. [181]	2019	121	Retrospective Study	Laparoscopic/Open
Göçmen et al. [85]	2010	22	Prospective Cohort Study	Robotic/Open
Goel et al. [100]	2011	97	Retrospective Study	Robotic/Open
Grabosch et al. [101]	2013	2	Case series	Laparoscopic
Graves et al. [102]	2012	760	Randomized Controlled Trial	Laparoscopic/Open
Gueli Alletti et al. [86]	2016	89	Retrospective Cohort Study	Laparoscopic/Robotic
Gunderson et al. [60]	2014	2596	Randomized Controlled Trial	Laparoscopic/Open
Helm et al. [88]	2011	168	Retrospective Study	Laparoscopic/Open
Herling et al. [89]	2016	360	Retrospective Cohort Study	Robotic/Open
Hinshaw et al. [90]	2016	136	Retrospective Cohort Study	Robotic/Open
Holloway et al. [91]	2009	100	Retrospective Study	Robotic
Holtz et al. [92]	2010	33	Retrospective Study	Laparoscopic/Robotic
Holub et al. [93]	2003	108	Prospective Cohort Study	Laparoscopic
Kalogiannidis et al. [135]	2007	169	Prospective Cohort Study	Laparoscopic (LAVH)/Open
Kroft et al. [137]	2015	12,104	Retrospective Cohort Study	Laparoscopic/Open
Kuoppala et al. [138]	2004	80	Retrospective Study	Laparoscopic/Open
Lau et al. [56]	2012	303	Retrospective Cohort Study	Robotic/Open
lavoue et al. [57]	2014	163	Retrospective Cohort Study	Robotic/Open
Lee et al. [105]	2014	105	Prospective Cohort Study	Laparoscopic
Lee et al. [106]	2016	287	Prospective Cohort Study	Laparoscopic
Lee et al. [66]	2008	35	Retrospective Study	Laparoscopic
Lee et al. [65]	2018	17,692	Retrospective Study	Laparoscopic
Lee et al. [108]	2016	9020	Retrospective Study	Laparoscopic/Robotic
Lee et al. [109]	2013	110	Randomized Controlled Trial	Laparoscopic with/without manipulator
Lee et al. [107]	2014	3	Prospective Study	NOTES surgery
Lee et al. [104]	2010	6	Retrospective Study	Robotic
Leiserowtz et al. [110]	2009	12,743	Retrospective Cohort Study	Laparoscopic (LAVH)/Open
Leitao et al. [111]	2013	475	Prospective Cohort Study	Laparoscopic/Robotic
Leitao et al. [112]	2012	752	Prospective Study	Laparoscopic/Robotic/Open
Leitao et al. [113]	2016	426	Retrospective Study	Laparoscopic/Robotic/Open/Vaginal
Li et al. [114]	2011	86	Retrospective Study	Laparoscopic/Open
Liang et al. [115]	2013	395	Retrospective Study	Robotic
Liauw et al. [117]	2003	30	Retrospective Study	Laparoscopic
Lim et al. [119]	2000	40	Retrospective Study	Laparoscopic (LAVH)/Open
Lim et al. [120]	2008	46	Retrospective Study	Laparoscopic with/without manipulator
Limbachiya et al. [121]	2020	88	Retrospective Study	Laparoscopic
Lindfors et al. [122]	2018	278	Retrospective Study	Robotic/Open
Lindfors et al. [123]	2020	217	Retrospective Study	Robotic/Open
Liu et al. [124]	2017	211	Retrospective Study	Laparoscopic/Open
Loaec et al. [125]	2018	20	Retrospective Study	Robotic
Lowe et al. [126]	2010	395	Retrospective Study	Robotic
Lowe et al. [127]	2009	405	Retrospective Study	Robotic
Lu et al. [128]	2013	272	Randomized Controlled Trial	Laparoscopic/Open
Lu et al. [129]	2012	238	Retrospective Study	Laparoscopic/Open
Lunde et al. [130]	2020	207	Nested Case Control Study	Robotic
Lundin et al. [131]	2020	49	Randomized Controlled Trial	Robotic/Open
Lundin et al. [132]	2019	50	Randomized Controlled Trial	Robotic/Open
Machida et al. [134]	2018	613	Retrospective Study	Laparoscopic/Open
Machida et al. [133]	2016	333	Case Control Study	Laparoscopic with cytology before and after manipulator
Mäenpää et al. [9]	2016	99	Prospective Cohort Study	Laparoscopic/Robotic
Malzoni et al. [170]	2009	159	Randomized Controlled Trial	Laparoscopic/Open
Peiretti et al. [219]	2009	80	Prospective Study	Robotic
Piovano et al. [140]	2014	271	Prospective study	Surgery/Radiotherapy
Praiss et al. [141]	2019	17,935	Retrospective Study	Minimally Invasive
Rabischong et al. [61]	2011	207	Retrospective Study	Laparoscopic
Rajadurai et al. [139]	2018	90	Retrospective Study	Laparoscopic/Robotic
Raventos-Tato [142]	2019	138	Retrospective Study	Laparoscopic/Robotic/Open
Roberts et al. [143]	2011	95	Retrospective Study	Laparoscopic (LAVH)/Laparoscopic (TLH)/Open
Rocha-Guevara et al. [144]	2015	17,935	Retrospective Study	Minimally Invasive
Safdieh et al. [145]	2017	43,985	Retrospective Study	Robotic/Open
Salehi et al. [146]	2018	120	Randomized Controlled Trial	Laparoscopic/Robotic
Sandadi et al. [147]	2012	573	Retrospective Study	Laparoscopic/Robotic
Santi et al. [148]	2010	240	Retrospective Study	Laparoscopic/Open
Scalici et al. [149]	2015	2076	Retrospective Study	Laparoscopic/Robotic
Scribner et al. [150]	1999	36	Retrospective Study	Laparoscopic/Open
Scribner et al. [151]	2001	125	Retrospective Study	Laparoscopic/Open
Seamon [59]	2009	79	Retrospective study	Robotic
Seamon et al. [152]	2009	181	Prospective/ retrospective Study	Laparoscopic/Robotic
Seracchioli [153]	2005	113	Retrospective Study	Laparoscopic/Open
Seror [154]	2014	146	Retrospective Study	Laparoscopic/Robotic
Siesto et al. [155]	2010	108	Retrospective Study	Laparoscopic/Open
Simpson et al. [156]	2020	4640	Retrospective Study	Laparoscopic/Laparoscopic (LAVH)/Robotic/Open
Singh et al. [157]	2017	9145	Retrospective Study	Laparoscopic
Slaughter et al. [158]	2014	380	Retrospective Study	Laparoscopic/Robotic
Sofer et al. [159]	2020	138	Retrospective Study	Robotic/Open
Soliman et al. [160]	2011	25	Retrospective Study	Laparoscopic
Somashekar et al. [166]	2014	50	Randomized Controlled Trial	Robotic/Open
Song et al. [161]	2020	135	Retrospective Study	Robotic/Open
Sonoda et al. [162]	2001	377	Retrospective Study	Laparoscopic (LAVH)/Open
Spencer et al. [163]	2012	133	Retrospective Study	Laparoscopic
Spirtos et al. [164]	1996	30	Retrospective Study	Laparoscopic/Open
Subramania et al. [165]	2011	73	Retrospective Study	Robotic
Tanaka et al. [167]	2020	913	Retrospective Study	Laparoscopic/Open
Tang et al. [168]	2012	239	Retrospective Cohort study	Robotic/Open
Taşkın et al. [169]	2012	153	Retrospective Study	Laparoscopic/Robotic/Open/Vaginal
Tinelli [171]	2014	75	Retrospective Study	Laparoscopic/Open
Tinelli et al. [217]	2011	226	Retrospective Study	Laparoscopic/Open
Togami et al. [172]	2020	155	Retrospective Study	Laparoscopic/Open
Tollund et al. [173]	2006	86	Retrospective Study	Laparoscopic (LAVH)/Laparoscopic (TLH)/Open
Tozzi et al. [175]	2005	122	Randomized Controlled Trial	Laparoscopic/Open
Turner et al. [87]	2015	335	Retrospective Study	Laparoscopic/Robotic
Turunen et al. [214]	2013	227	Retrospective Study	Laparoscopic/Robotic
Uccella et al. [176]	2016	1266	Retrospective Study	Laparoscopic/Open
Uccella et al. [177]	2016	1606	Retrospective Study	Laparoscopic/Open
Ulm et al. [178]	2016	325	Retrospective Study	Robotic/Open
Vardar et al. [180]	2019	801	Retrospective Study	Laparoscopic/Open
Venkat et al. [182]	2012	54	Retrospective Study	Robotic/Open
Walker et al. [183]	2012	2181	Randomized Controlled Trial	Laparoscopic/Open
Wong et al. [184]	2005	64	Retrospective Study	Laparoscopic/Open
Wright et al. [186]	2012	8018	Retrospective Study	Laparoscopic/Open
Wright et al. [185]	2016	6304	Retrospective Study	Laparoscopic/Robotic/Open
Xu et al. [187]	2020	81	Prospective Observational Study	Laparoscopic/Open
Yin et al. [188]	2015	32	Retrospective Study	Laparoscopic
Yu et al. [189]	2013	2247	Retrospective Study	Laparoscopic/Robotic/Open
Zakhari et al. [190]	2015	10,347	Retrospective Study	Laparoscopic/Robotic
Zapico et al. [191]	2005	90	Retrospective Study	Laparoscopic/Open
Zhang et al. [192]	2014	458	Retrospective Study	Minimally Invasive/Open
Zorlu et al. [193]	2005	52	Randomized Controlled Trial	Laparoscopic/Open
Zullo et al. [194]	2005	84	Prospective long-term extension study	Laparoscopic/Open

**Table 2:** League table showing pairwise comparisons among the three surgical techniques (LRS: laparoscopic surgery, OS: open surgery, RS: robotic surgery) for the 14 outcomes from the Bayesian network meta-analysis. Rows represent the references, and columns represent the comparators. 95% credible intervals are included in parentheses. The clinically significant differences are shown in bold.

Outcome		OS	LRS	RS
**Blood Loss**	**OS**	0 (0, 0)	**−226.90** **(−298.40, −155.90)**	**−257.20** **(−351.20, −163.80)**
	**LRS**	226.90 (155.90, 298.40)	0 (0, 0)	−30.33 (−122.20, 61.62)
	**RS**	257.20 (163.80, 351.20)	30.33 (−61.62, 122.2)	0 (0, 0)
**Duration of Operation**	**OS**	0 (0, 0)	**18.95** **(7.68, 30.20)**	**29.00** **(13.66, 44.23)**
	**LRS**	−18.95 (−30.20, −7.68)	0 (0, 0)	10.05 (−5.60, 25.48)
	**RS**	−29.00 (−44.22, −13.66)	−10.05 (−25.48, 5.60)	0 (0, 0)
**Length of Stay in Hospital**	**OS**	0 (0, 0)	**−3.54** **(−4.22, −2.87)**	**−3.79** **(−4.79, −2.79)**
	**LRS**	3.54 (2.87, 4.22)	0 (0, 0)	−0.25 (−1.26, 0.77)
	**RS**	3.79 (2.79, 4.79)	0.25 (−0.77, 1.26)	0 (0, 0)
**Total Lymph Nodes**	**OS**	0 (0, 0)	0.40 (−1.18, 2.01)	−0.06 (−2.72, 2.69)
	**LRS**	−0.40 (−2.01, 1.18)	0 (0, 0)	−0.46 (−2.91, 2.09)
	**RS**	0.06 (−2.69, 2.72)	0.46 (−2.09, 2.91)	0 (0, 0)
**Blood Transfusion**	**OS**	1 (1, 1)	**0.30** **(0.19, 0.48)**	**0.26** **(0.12, 0.53)**
	**LRS**	3.32 (2.09, 5.38)	1 (1, 1)	0.85 (0.4, 1.79)
	**RS**	3.90 (1.89, 8.31)	1.17 (0.56, 2.50)	1 (1, 1)
**Fever**	**OS**	1 (1, 1)	**0.57** **(0.30, 0.98)**	0.42 (0.07, 2.21)
	**LRS**	1.75 (1.02, 3.29)	1 (1, 1)	0.74 (0.11, 4.51)
	**RS**	2.37 (0.45, 14.38)	1.35 (0.22, 8.73)	1 (1, 1)
**Infection**	**OS**	1 (1, 1)	**0.50** **(0.28, 0.93)**	0.84 (0.35, 2.01)
	**LRS**	1.99 (1.07, 3.60)	1 (1, 1)	1.66 (0.69, 3.99)
	**RS**	1.20 (0.50, 2.84)	0.60 (0.25, 1.46)	1 (1, 1)
**Ileus**	**OS**	1 (1, 1)	**0.46** **(0.29, 0.68)**	**0.18** **(0.08, 0.41)**
	**LRS**	2.16 (1.47, 3.40)	1 (1, 1)	**0.40** **(0.17, 0.87)**
	**RS**	5.44 (2.43, 12.93)	2.50 (1.14, 5.74)	1 (1, 1)
**VTE**	**OS**	1 (1, 1)	0.57 (0.36, 1.10)	0.80 (0.33, 1.86)
	**LRS**	1.75 (0.91, 2.79)	1 (1, 1)	1.39 (0.55, 2.91)
	**RS**	1.26 (0.54, 3.01)	0.72 (0.34, 1.83)	1 (1, 1)
**Disease-free Survival**	**OS**	1 (1, 1)	1.35 (0.80, 2.32)	**3.29** **(1.46, 8.36)**
	**LRS**	0.74 (0.43, 1.26)	1 (1, 1)	**2.45** **(1.04, 6.34)**
	**RS**	0.30 (0.12, 0.69)	0.41 (0.16, 0.97)	1 (1, 1)
**Recurrence**	**OS**	1 (1, 1)	**0.64** **(0.47, 0.84)**	0.64 (0.35, 1.19)
	**LRS**	1.57 (1.20, 2.15)	1 (1, 1)	1.02 (0.55, 1.95)
	**RS**	1.55 (0.84, 2.86)	0.98 (0.51, 1.81)	1 (1, 1)
**Total Complications**	**OS**	1 (1, 1)	**0.38** **(0.29, 0.51)**	**0.34** **(0.22, 0.51)**
	**LRS**	2.61 (1.97, 3.45)	1 (1, 1)	0.88 (0.58, 1.31)
	**RS**	2.97 (1.98, 4.55)	1.14 (0.76, 1.73)	1 (1, 1)
**Total Intra-operative Complications**	**OS**	1 (1, 1)	1.04 (0.75, 1.49)	**0.39** **(0.18, 0.78)**
	**LRS**	0.96 (0.67, 1.33)	1 (1, 1)	**0.38** **(0.17, 0.75)**
	**RS**	2.55 (1.28, 5.47)	2.66 (1.34, 5.79)	1 (1, 1)
**Total Post-operative Complications**	**OS**	1 (1, 1)	**0.48** **(0.34, 0.70)**	**0.46** **(0.27, 0.78)**
	**LRS**	2.07 (1.43, 2.98)	1 (1, 1)	0.95 (0.54, 1.63)
	**RS**	2.19 (1.29, 3.71)	1.05 (0.61, 1.84)	1 (1, 1)

## Data Availability

All data are available to share, either in the material submitted with the manuscript or upon request from the authors.
